# Miscellanea

**Published:** 1855-07

**Authors:** 


### MISCELLANEA.

Editorial Changes.—The Medical Society of Virginia has disposed of its interest in the Stethoscope. This periodical is now edited by Drs. Goodridge, A. Wilson, and Richmond A. Lewis.—Dr. C. A. Griswold has become associated with Dr. Reese in the editorial management of the American Medical Gazette.—The British and Foreign Medico-Chirurgical Review will hereafter be conducted by Dr. Sieveking, Professor Parkes having vacated the editorial chair in consequence of his appointment as Superintendent of the English New Civil Hospital in the East.—The Edinburgh Medical and Sugical Journal, and the Monthly Journal of Medical Science, have amalgamated, Prof. Bennet retiring from his editorial labors.—Dr. R. Grundy has become associated with Dr. Dawson, of Columbus, Ohio, as assistant editor of the Ohio Medical and Surgical Journal.

Professional Changes.—Dr. Gibson, on resigning the post which he had occupied in the University of Pennsylvania for thirty-seven years, was appointed by the Trustees Emeritus Professor of Surgery.

Dr. Horace Green has resigned the chair of Theory and Practice of Medicine in the New York Medical College, and Dr. Henry G. Cox, Physician in Chief of the State Emigrant Hospital, has been appointed in his stead. Dr. Green becomes *Emeritus* Professor, will continue to be President of the Faculty, and to lecture on Diseases of the Air Passages. In the same school Dr. Peaslee and Dr. Parker exchange chairs, the former taking Physiology and Pathology, and the latter Anatomy.

Dr. Brown Séquard, about whom there was such a flourish of trumpets a year ago, has resigned the Professorship which he held in the Medical College of Virginia and returned to France. Dr. Levin S. Jones, of Accomac County, Virginia, has been elected his successor. Dr. A. E. Peticolas, of Richmond, has been chosen Professor of Anatomy in the same Institution to fill the vacancy occasioned by the death of Dr. Johnson.

It is not Dr. W. M. Boling, of Montgomery, Ala., who is Professor in the Atlanta College, Georgia.—Dr. Buchanan, one of the Faculty of the Nashville Medical School, has been appointed to the chair of Anatomy in the Atlanta School.

Two vacancies have recently occurred in the Rush Medical College, Chicago. Applicants may address Dr. Brainard, Chicago.

Dr. La Roche has declined the Professorship offered him in the Memphis Medical College.

Handsome.—The French government has presented Surgeon Williamson, U. S. N., and Dr. James Harrison, with gold medals as acknowledgments of their humane services to the steamer Chimére, at Norfolk, during the prevalence of yellow fever on board.—The last class to which Prof. Gibson lectured intend placing a portrait of the veteran teacher in the Wistar museum.—Prof. Austin Flint's friends in Buffalo have had an excellent lithograph of this gentleman taken, a copy of which has been sent to each of the subscribers of the Buffalo Medical and Sugical Journal, of which Dr. Flint was editor from 1845 to 1855.—A verdict of $1,500 has been recently rendered in the Supreme Court against an irregular practitioner of Boston, in an action for malicious prosecution.

The cholera was declared by the Board of Health of New Orleans to be epidemic in that city a short time ago. They have since stated that it had almost entirely disappeared. The disease is prevailing very generally and with much fatality on the Upper Mississippi river and its tributaries.—Lightning struck a lady in Pennsylvania a short time since; the fluid burnt the hair from the crown of her head, melted her hair-pins and left its mark down her body to where it passed through the floor. The lady lived through it, and is rapidly recovering.—A Retreat for the Insane has been established by Dr. Mead, of Cincinnati, near that city.—A sad mistake was made a few weeks ago by a druggist of New York, who put up emetic tartar for *pulvis antimonialis*. Its administration proved fatal.—Of Dr. W. L. Atlee's thirty cases of ovariotomy published in the Medical Examiner, thirteen terminated fatally, either from excessive hemorrhage or peritonitis.—Prof. Willard Parker, of New York, reports three cases of adult females in which he successfully removed vesical calculi by the suprapubic operation.—Scurvy has been prevailing to a distressing extent among the pauper population of this city, and along the lines of unfinished railway connecting here.—Dr. Knapp, of Cincinnati, maintains the identity of cholera and scurvy. If his theory be correct, and of this he emphatically declares there cannot be the shadow of a doubt, it is somewhat singular that the latter malady has not yet made its appearance in this vicinity.—It is stated on the authority of a surgeon in the U. S. Navy that there is in a church in Bremen a vault the atmosphere of which possesses the peculiar property of preserving from decay all bodies that may be placed therein.—A Cincinnati paper gives a narrative of the discovery of "some very curious petrified human bodies," found in Pennsylvania in the bed of one of the branches of the Alleghany river. The remains are supposed to be those of a man and woman. The feet of the man are composed of coal, his body of sand-stone, and his head of quartz and gneiss. The gentleman is supposed to have been buried with his head downwards, "and at just such depth that his head came in the gneiss, his body in the sand-stone formation, and his feet in a coal-bed." This knocks the whole science of geology into a cocked hat, if true.—Mr. Avery, for many years surgeen to the Charity Cross Hospital, London, died a short time since.—Dr. Courtenay S. King, of Charleston, S. C., aged twenty-four, and Dr. Isaac Draper, jr., of Massachusetts, both American Surgeons in the Russian service, died recently in the Crimea.—The case of Dr. Beale, of Philadelphia, is to be tried over on a writ of error.

